# Mitochondria-Derived Vesicles and Inflammatory Profiles of Adults with Long COVID Supplemented with Red Beetroot Juice: Secondary Analysis of a Randomized Controlled Trial

**DOI:** 10.3390/ijms26031224

**Published:** 2025-01-30

**Authors:** Emanuele Marzetti, Hélio José Coelho-Júnior, Riccardo Calvani, Giulia Girolimetti, Riccardo Di Corato, Francesca Ciciarello, Vincenzo Galluzzo, Clara Di Mario, Barbara Tolusso, Luca Santoro, Ottavia Giampaoli, Alberta Tomassini, Walter Aureli, Matteo Tosato, Francesco Landi, Cecilia Bucci, Flora Guerra, Anna Picca

**Affiliations:** 1 Fondazione Policlinico Universitario “Agostino Gemelli” IRCCS, 00168 Rome, Italy; emanuele.marzetti@policlinicogemelli.it (E.M.); coelhojunior@hotmail.com.br (H.J.C.-J.); francesca.cicciarello@policlinicogemelli.it (F.C.); vincenzo.galluzzo@policlinicogemelli.it (V.G.); clara.dimario@policlinicogemelli.it (C.D.M.); barbara.tolusso@policlinicogemelli.it (B.T.); luca.santoro@policlinicogemelli.it (L.S.); matteo.tosato@policlinigogemelli.it (M.T.); francesco.landi@unicatt.it (F.L.); picca@lum.it (A.P.); 2 Department of Geriatrics, Orthopedics and Rheumatology, Università Cattolica del Sacro Cuore, 00168 Rome, Italy; 3 Department of Experimental Medicine, University of Salento, Via Provinciale Lecce-Monteroni, 73100 Lecce, Italy; giulia.girolimetti@unisalento.it (G.G.); cecilia.bucci@unisalento.it (C.B.); 4 Institute for Microelectronics and Microsystems (IMM), CNR, Via Provinciale Lecce-Monteroni, 73100 Lecce, Italy; riccardo.dicorato@cnr.it; 5 Center for Biomolecular Nanotechnologies, Istituto Italiano di Tecnologia, 73010 Arnesano, Italy; 6 Department of Environmental Biology, Sapienza University of Rome, Piazzale Aldo Moro 5, 00185 Rome, Italy; ottavia.giampaoli@uniroma1.it; 7 NMR-Based Metabolomics Laboratory (NMLab), Sapienza University of Rome, 00185 Rome, Italy; 8 R&D, Aureli Mario S.S. Agricola, Via Mario Aureli 7, 67050 Ortucchio, Italy; tomassinialberta@gmail.com (A.T.); produzione@aurelimario.com (W.A.); 9 Department of Biological and Environmental Sciences and Technologies, Università del Salento, 73100 Lecce, Italy; flora.guerra@unisalento.it; 10 Department of Medicine and Surgery, LUM University, Str. Statale 100 Km 18, 70010 Casamassima, Italy

**Keywords:** cell quality, cytokine, extracellular vesicles, functional food, inflammation, mitochondrial DNA, muscle, physical performance

## Abstract

In a recent clinical trial, beetroot juice supplementation for 14 days yielded positive effects on systemic inflammation in adults with long COVID. Here, we explored the relationship between circulating markers of mitochondrial quality and inflammation in adults with long COVID as well as the impact of beetroot administration on those markers. We conducted secondary analyses of a placebo-controlled randomized clinical trial testing beetroot juice supplementation as a remedy against long COVID. Analyses were conducted in 25 participants, 10 assigned to placebo (mean age: 40.2 ± 11.5 years, 60% women) and 15 allocated to beetroot juice (mean age: 38.3 ± 7.7 years, 53.3% women). Extracellular vesicles were purified from serum by ultracentrifugation and assayed for components of the electron transport chain and mitochondrial DNA (mtDNA) by Western blot and droplet digital polymerase chain reaction (ddPCR), respectively. Inflammatory markers and circulating cell-free mtDNA were quantified in serum through a multiplex immunoassay and ddPCR, respectively. Beetroot juice administration for 14 days decreased serum levels of interleukin (IL)-1β, IL-8, and tumor necrosis factor alpha, with no effects on circulating markers of mitochondrial quality control. Significant negative associations were observed between vesicular markers of mitochondrial quality control and the performance on the 6 min walk test and flow-mediated dilation irrespective of group allocation. These findings suggest that an amelioration of mitochondrial quality, possibly mediated by mitochondria-derived vesicle recycling, may be among the mechanisms supporting improvements in physical performance and endothelial function during the resolution of long COVID.

## 1. Introduction

The recovery from COVID-19 is unpredictable and contingent upon the severity of acute disease, pre-existing comorbidities, and age [1]. As per the World Health Organization (WHO), full recovery from COVID-19 requires approximately two weeks in moderate cases and three to six weeks in more severe infections [2]. Nevertheless, a significant number of individuals experience COVID-19-related symptoms for weeks or months after viral clearance [3,4], a condition referred to as post-COVID condition or long COVID [5].

Cough, fever, dyspnea, fatigue, gastrointestinal and musculoskeletal complaints (e.g., myalgia, joint pain), and anosmia/dysgeusia are among the most prevalent long-term signs and symptoms of COVID-19 [3,4]. Furthermore, declines in muscle strength [6] and endothelial dysfunction [7] are frequent findings among adults with long COVID, with a negative impact on symptom persistence (e.g., fatigue) and overall quality of life [8]. Indeed, sarcopenia, identified according to international guidelines, is more frequent in individuals with long COVID than in the general population regardless of age [6]. This observation has led to hypothesizing that sarcopenia may be the biological substratum of long COVID-associated fatigue and weakness [6].

Nutraceuticals, bioactive foods, and dietary supplements have been tested for their effectiveness against long COVID [9]. Acute beetroot juice supplementation conveys beneficial effects on cardiovascular health, exercise performance, pulmonary function, metabolic homeostasis, and inflammation [10,11,12,13]. The health benefits conveyed by red beetroot juice have been mostly attributed to its high nitrate content. The ingestion of nitrate-rich foods increases nitric oxide (NO) availability via the nitrate–nitrite–NO pathway [14]. NO bioavailability positively modulates mitochondrial respiration, reduces the oxygen cost of exercise, and enhances muscular and brain perfusion [15,16]. Additional bioactive ingredients (e.g., polyphenols, carotenoids, betalains, organic acids) with antioxidant and anti-inflammatory properties are contained in beetroot juice [11]. Among these substances, betalains are a class of natural pigments including the red-violet betacyanins and the yellow-orange betaxanthins that can stimulate the activity of antioxidant enzymes and blunt inflammation [17].

Altogether, these properties make beetroot juice an ideal candidate for the management of long COVID. However, in a recent clinical trial, beetroot juice supplementation for 14 days failed to improve functional outcomes in adults with long COVID [18]. Yet, a positive effect on systemic inflammation was observed following beetroot juice supplementation. Previous studies have shown a relationship between circulating markers of cellular/mitochondrial quality and inflammation and physical performance [19,20,21]. Defective mitochondrial quality control (MQC) processes can incite systemic inflammation also through the unloading of molecules, including mitochondrial DNA (mtDNA), holding proinflammatory properties [22]. A persistent activation of the inflammatory response characterizes individuals with long COVID [23]. However, little is known about the molecular triggers of this response. In addition, whether a relationship exists between cell and MQC markers and inflammatory profiles in adults with long COVID is presently unknown. Neither is empirical evidence available on the possible impact of beetroot juice administration on those markers in individuals with long COVID.

To address these research questions, we conducted secondary analyses of a randomized controlled trial (RCT) to explore the associations between markers of MQC and inflammation and measures of physical performance and endothelial function in adults with long COVID supplemented with red beetroot juice or receiving placebo.

## 2. Results

### 2.1. Characteristics of Study Participants

Analyses were conducted in 25 participants, 10 assigned to placebo (four men and six women; mean age: 40.2 ± 11.5 years) and 15 allocated to red beetroot juice (seven men and eight women; mean age: 38.3 ± 7.7 years). The main baseline characteristics of study participants according to group assignment are listed in Table 1.

The two groups did not differ for any parameter. Two participants allocated to beetroot juice were hospitalized once during the acute COVID-19 episode. After 14 days of intervention, the 6 min walk test (6MWT) distance was 585.0 ± 81.7 m (delta vs. baseline: 25.7 ± 27.0 m) in the placebo group and 585.0 ± 52.0 m (delta vs. baseline: 13.4 ± 48.6 m) in the beetroot juice group. Flow-mediated dilation (FMD) after 14 days of intervention was 13.0 ± 5.1% (delta vs. baseline: 0.4 ± 7.8%) in the placebo group and 13.2 ± 2.8% (delta vs. baseline: 1.3 ± 3.4%) in the beetroot juice group.

### 2.2. Effects of Interventions on Inflammatory and Vesicular/Mitochondrial Markers

**Figure 1:**
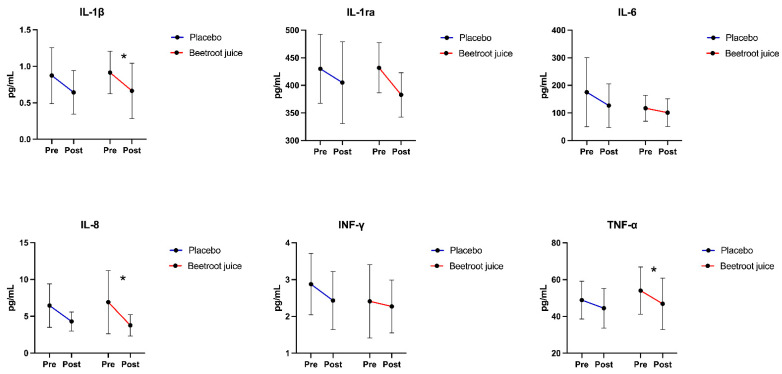
Changes from baseline to day 14 in serum concentrations of inflammatory markers in the placebo (*n* = 10) and beetroot juice (*n* = 15) groups. Abbreviations: IFN-γ, interferon gamma; IL, interleukin; IL-1ra, interleukin 1 receptor antagonist; and TNF-α, tumor necrosis factor alpha. * *p* < 0.05 vs. baseline.

Serum levels of the proinflammatory markers interleukin (IL)-1β, IL-8, and tumor necrosis factor alpha (TNF-α) were significantly reduced after 14 days of intervention in participants receiving red beetroot juice (Figure 1). No other significant pre/post-intervention differences were observed in either intervention group.

**Figure 2:**
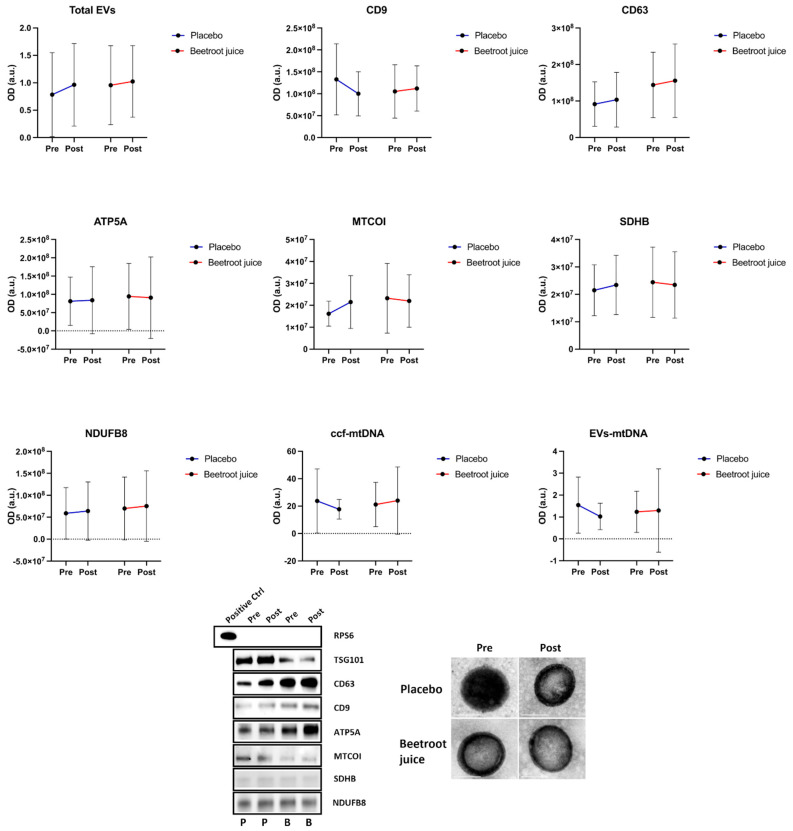
Changes from baseline to day 14 in vesicular/mitochondrial markers in the placebo (*n* = 10) and beetroot juice (*n* = 15) groups. The lower panels show representative Western blot bands and transmission electron microscope images of extracellular vesicles. Abbreviations: ATP5A, ATP synthase F1 subunit alpha; a.u., arbitrary unit; B, beetroot juice; ccf-mtDNA, circulating cell-free mitochondrial DNA; CD, cluster of differentiation; Ctrl, control; EVs, extracellular vesicles; MTCOI, mitochondrial cytochrome C oxidase I; OD, optical density; NDUFB8, NADH:ubiquinone oxidoreductase subunit B8; RPS6, ribosomal protein s6; and SDHB, succinate dehydrogenase [ubiquinone] iron–sulfur subunit.

Likewise, no significant changes were observed for any vesicular/mitochondrial markers in either intervention groups (Figure 2).

**Figure 3:**
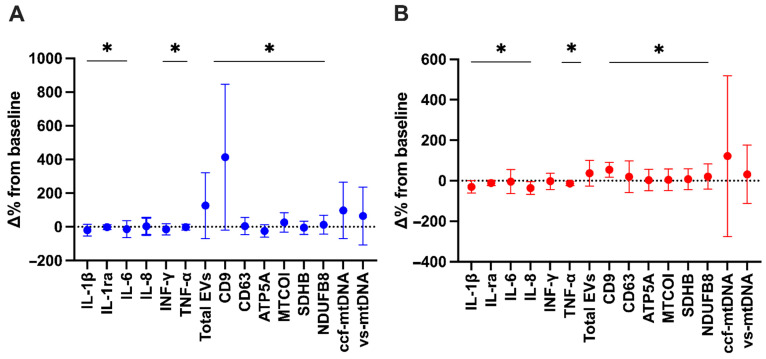
Changes from baseline to day 14 in inflammatory and vesicular/mitochondrial markers adjusted for pre-intervention values in (**A**) placebo (*n* = 10) and (**B**) beetroot juice (*n* = 15) groups. Abbreviations: ATP5A, ATP synthase F1 subunit alpha; CD, cluster of differentiation; ccf-mtDNA, circulating cell-free mitochondrial DNA; EVs, extracellular vesicles; IL, interleukin; IL-1ra, interleukin 1 receptor antagonist; MTCOI, mitochondrial cytochrome C oxidase I; NDUFB8, NADH:ubiquinone oxidoreductase subunit B8; SDHB, succinate dehydrogenase [ubiquinone] iron–sulfur subunit; and vs-mtDNA, vesicular mitochondrial DNA. * *p* < 0.05 for comparisons between pre- and post-intervention adjusted for baseline values according to analysis of covariance (ANCOVA).

The analysis of changes from baseline to day 14 of inflammatory and vesicular/mitochondrial markers adjusted for pre-intervention values showed significant differences in both intervention groups. Most inflammatory markers were different from baseline in participants who received placebo (IL-1β, IL-ra, IL-6, interferon gamma (IFN-γ), and tumor necrosis factor alpha (TNF-α); *p* < 0.05) (Figure 3A) and those assigned to beetroot juice (IL-1β, IL-ra, IL-6, IL-8, and TNF-α; *p* < 0.05) (Figure 3B).

As per the vesicular/mitochondrial markers, most of them were different from baseline in both participant groups (cluster of differentiation (CD) 9, CD63, ATP synthase F1 subunit alpha (ATP5A), mitochondrial cytochrome C oxidase I (MTCOI), NADH:ubiquinone oxidoreductase subunit B8 (NDUFB8), and succinate dehydrogenase [ubiquinone] iron–sulfur subunit (SDHB); *p* < 0.05) (Figure 3B). Results of two-way analysis of covariance (ANCOVA) showed no significant differences between placebo and beetroot juice groups for post-intervention values adjusted for baseline data (Figure 3A,B).

### 2.3. Relationship Between Inflammatory and Vesicular/Mitochondrial Markers and Functional Measures

**Figure 4:**
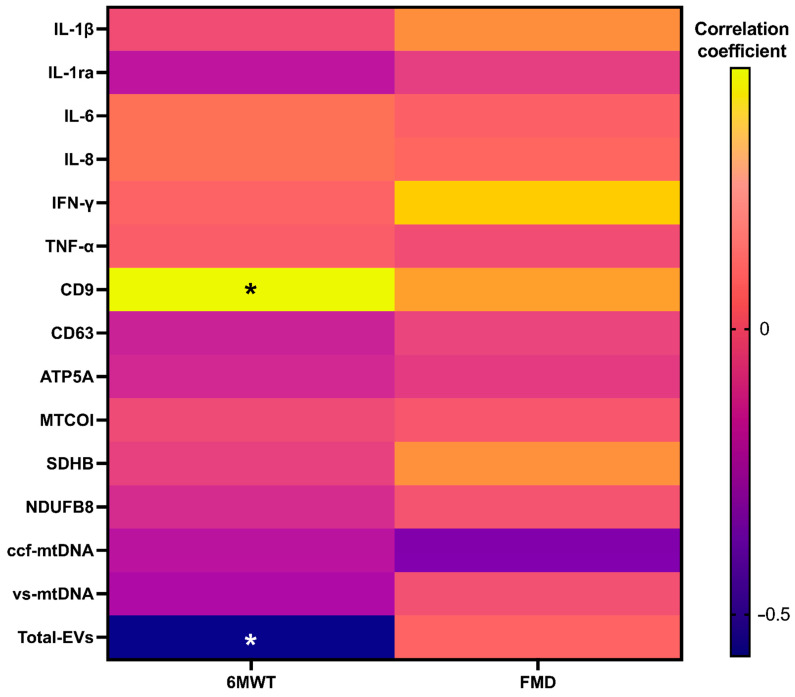
Correlation analyses between inflammatory and vesicular/mitochondrial markers and measures of physical and endothelial function at 14 days in the whole study sample (*n* = 25). Abbreviations: ATP5A, ATP synthase F1 subunit alpha; CD, cluster of differentiation; ccf-mtDNA, circulating cell-free mitochondrial DNA; EVs, extracellular vesicles; FMD, flow-mediated dilation; IFN-γ, interferon gamma; IL, interleukin; IL-1ra, interleukin 1 receptor antagonist; MTCOI, mitochondrial cytochrome C oxidase I; NDUFB8, NADH:ubiquinone oxidoreductase subunit B8; SDHB, succinate dehydrogenase [ubiquinone] iron–sulfur subunit; and vs-mtDNA, vesicular mitochondrial DNA. * *p* < 0.05.

Results of correlation analyses between inflammatory and vesicular/mitochondrial markers and measures of physical and endothelial function in the whole study sample at baseline indicated a positive association between the performance on the 6MWT and circulating levels of vesicular CD9 (*p* = 0.028). A negative association was identified between the performance on the 6MWT and total EV levels (*p* = 0.004) (Figure 4).

**Figure 5:**
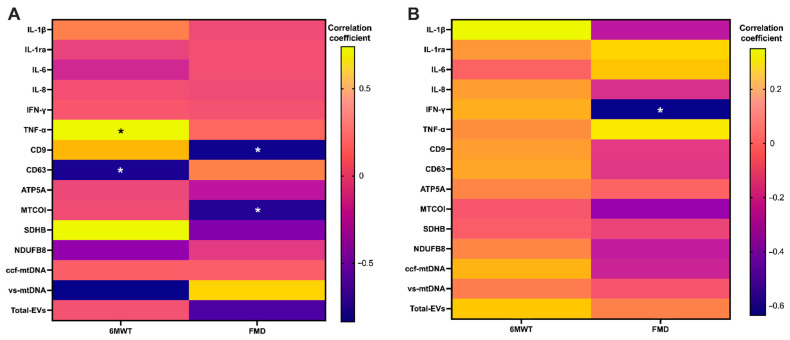
Correlation analyses between pre/post-intervention differences in inflammatory and vesicular/mitochondrial markers and measures of physical and endothelial function at 14 days in (**A**) the placebo group (*n* = 10) and (**B**) participants assigned to beetroot juice (*n* = 15). Abbreviations: ATP5A, ATP synthase F1 subunit alpha; CD, cluster of differentiation; ccf-mtDNA, circulating cell-free mitochondrial DNA; EVs, extracellular vesicles; IFN-γ, interferon gamma; IL, interleukin; IL-1ra, interleukin 1 receptor antagonist; MTCOI, mitochondrial cytochrome C oxidase I; NDUFB8, NADH:ubiquinone oxidoreductase subunit B8; and SDHB, succinate dehydrogenase [ubiquinone] iron–sulfur subunit; vs-mtDNA, vesicular mitochondrial DNA. * *p* < 0.05.

Results of correlation analyses of pre/post-intervention changes adjusted by groups indicated specific associations according to the intervention. In participants who received placebo, a positive association was observed between 6MWT at 14 days and TNF-α levels (*p* = 0.032), whereas CD63 showed a negative association (*p* = 0.021). Negative associations were also found between FMD and CD9 and vesicular levels of MTCOI (*p* = 0.034 and *p* = 0.048, respectively) (Figure 5A). Participants assigned to beetroot juice supplementation showed negative associations between FMD at 14 days and IFN-γ (*p* = 0.030) (Figure 5B).

**Figure 6:**
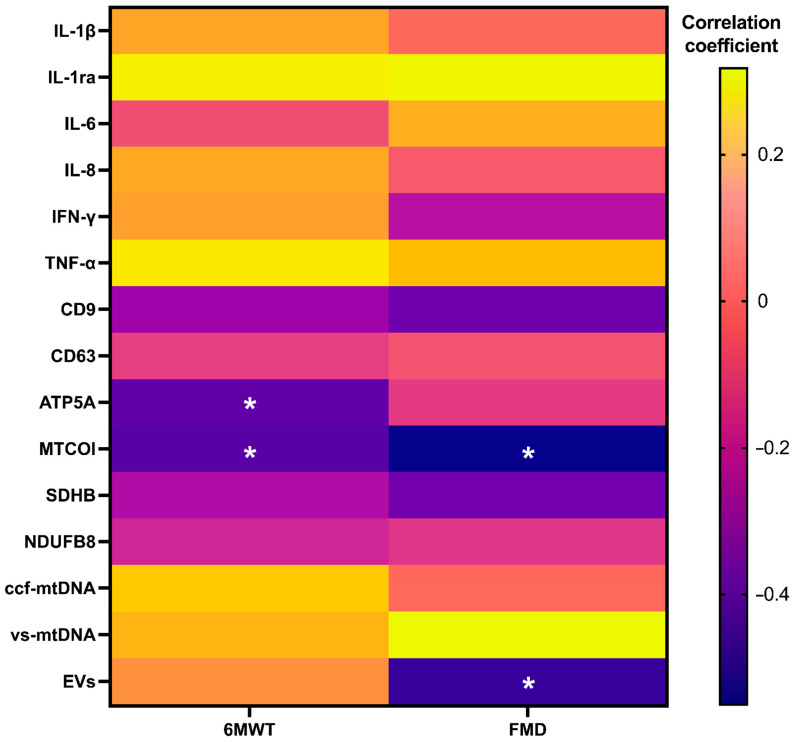
Correlation analyses between pre/post-intervention differences in inflammatory and vesicular/mitochondrial markers and measures of physical and endothelial function at 14 days independent of intervention group (*n* = 25). Abbreviations: ATP5A, ATP synthase F1 subunit alpha; CD, cluster of differentiation; ccf-mtDNA, circulating cell-free mitochondrial DNA; EVs, extracellular vesicles; IFN-γ, interferon gamma; IL, interleukin; IL-1ra, interleukin 1 receptor antagonist; MTCOI, mitochondrial cytochrome C oxidase I; NDUFB8, NADH:ubiquinone oxidoreductase subunit B8; SDHB, succinate dehydrogenase [ubiquinone] iron–sulfur subunit; vs-mtDNA, vesicular mitochondrial DNA * *p* < 0.05.

Finally, results of correlation analyses between inflammatory and vesicular/mitochondrial markers and measures of physical and endothelial function independent of intervention group indicated a negative and significant association of vesicular levels of ATP5A (*p* = 0.038) and MTCOI (*p* = 0.032), with 6MWT performance at 14 days and MTCOI (*p* = 0.018), and of total EV content (*p* = 0.037) with FMD at 14 days (Figure 6).

## 3. Discussion

Through secondary analyses of an RCT, we found that the administration of red beetroot juice for 14 days was associated with a decrease in serum levels of IL-1β, IL-8, and TNF-α in adults with long COVID (Figure 1), with no effects on circulating markers of MQC (Figure 2). After adjustment for baseline values, no significant differences between the two intervention groups were observed for any assayed marker. However, group-specific relationships between circulating markers and measures of physical and endothelial function were identified. In participants assigned to beetroot juice supplementation, IFN-γ was negatively and significantly associated with FMD (Figure 5B). Irrespective of group allocation, significant negative associations were observed between circulating EV-associated ATP5A and MTCOI and the performance on the 6MWT and between EV-associated MTCOI and circulating levels of EVs and FMD (Figure 6).

Long COVID has a complex pathophysiology encompassing inflammatory and autoimmune processes, perturbations in metabolic pathways, and alterations in endothelial function and redox homeostasis [24]. Several bioactive compounds, including flavonoids, and aromatic and sulfur compounds have been tested as possible remedies for long COVID [9]. Compounds that can feed the nitrate–nitrite–NO pathway have also been tested. 

Bioactive NO in mammals is classically produced from l-arginine and oxygen by a family of enzymes, known as NO synthases (NOSs). Inorganic nitrate from dietary sources, including beetroot juice, is rapidly absorbed in the small intestine and can also increase NO availability through the nitrate–nitrite–NO pathway [14]. While most circulating nitrate is excreted with urine, up to 25% is actively transported in salivary glands and concentrated in saliva [25]. Via the activity of nitrate reductase enzymes, commensal facultative anaerobic bacteria reduce nitrate to nitrite in the mouth. Nitrate conversion into nitrite is necessary for mammalian cells to metabolize this ion. Nitrite can be decomposed into NO and other bioactive nitrogen oxides in the acidic environment of the stomach [26], which regulates relevant physiological functions. Residual nitrate and nitrite are absorbed from the intestine and delivered into the circulation where they can be converted into bioactive NO via the activity of several enzymes (e.g., xanthine oxidoreductase, mitochondrial electron transport chain enzymes) and pathways in the setting of hypoxia and acidosis, such as that of exercising muscles [12].

Beetroot juice contains also betalains, bioactive ingredients with a dual role in inhibiting inflammation and stimulating antioxidant enzymes activity [11,27]. Betanin, the most abundant betalain contained in beetroots, may blunt inflammation via the inhibition of cyclooxygenase 2 [28] and lipoxygenase 1 activity [17], as well as nuclear factor κB (NF-κB) signaling [29]. Moreover, betanin is a reactive oxygen species (ROS) scavenger [30] able to enhance the expression of catalase, superoxide dismutase, and glutathione peroxidase via activation of the nuclear factor erythroid 2-related factor 2 (Nrf2)/antioxidant response element (ARE) pathway [31]. Altogether, these effects may support mitochondrial and cellular quality control processes that would otherwise fuel systemic inflammation via the unloading of proinflammatory molecules, including mtDNA [22].

ccf-mtDNA is released from dead cells/tissues as well as by mitochondria for signaling purposes. Increasing levels of ccf-mtDNA and/or mitochondria-derived vesicles have been described as a mechanism that may be modulated by the severity of mitochondrial damage and an alternative route to mitophagy [32,33]. This pathway of mitochondrial disposal is triggered in the setting of mild mitochondrial damage and ROS production [34,35]. Mitochondrial clearance via EVs along the endosomal pathway has also been reported in the setting of lysosomal pathway inhibition [36]. In both cases, cells can selectively guide the packaging of mitochondrial constituents into EVs to achieve MQC and avoid the release of EV-free, damaged mitochondrial constituents [37]. The latter incite innate immunity because of their nature of proinflammatory damage-associated molecular patterns (DAMPs) [37]. In line with this view, our findings of a negative correlation between vesicular ATP5A and MTCOI and the performance on the 6MWT and between vesicular MTCOI and circulating EVs and FMD measures irrespective of group allocation provide interesting clues on the mechanisms of long COVID resolution. Although a causal relationship cannot be inferred, these data, together with the observation of a general quenching of the inflammatory response, support a relationship between the amelioration of mitochondrial quality, possibly related to recycling through mitochondria-derived vesicles, and improvements in physical performance and endothelial function. This piece of information is in line with recent findings showing that an altered MQC and systemic inflammation are associated with declines in muscle power [38].

Although our investigation reports novel findings on the possible role of cell quality processes in long COVID, this study has limitations that deserve to be acknowledged. First, the small sample size and the pilot nature of this study did not allow performing analysis according to sex. Further to this, general linear models were only adjusted for pre-intervention values. Future studies controlling for other possible covariates are needed. Second, the short duration of the intervention (two weeks vs. the recommended four to six weeks) as well as the timing of data collection (assessment of beetroot juice effects in the non-acute phase) may have hampered a full appreciation of the intervention effects. Third, although participants were asked to refrain from consuming foods rich in inorganic nitrate for at least 12 h prior to each study visit, the nitrate intake could not be used as a confounder in the analysis. Similarly, differences in lifestyle habits, including physical activity levels, and the consumption of substances with vasoactive properties were not accounted for.

## 4. Materials and Methods

### 4.1. Study Design and Participants 

The present study involves secondary analyses of a single-center, single-blinded, placebo-controlled randomized trial conducted at the post-acute COVID-19 outpatient clinic of the Fondazione Policlinico A. Gemelli IRCCS (Rome, Italy) from March 2021 to July 2021 [14]. The study protocol was approved by the Ethics Committee of the Università Cattolica del Sacro Cuore (Rome, Italy) (Prot. no. 0013008/20), and the trial was registered in ClinicalTrials.gov (NCT06535165). All participants provided written informed consent prior to enrolment.

Participant selection criteria are reported elsewhere [14]. Briefly, candidate participants were eligible for inclusion if they were 20 to 60 years old, had a previous SARS-CoV-2 infection, had a negative COVID-19 swab test at least four weeks prior to trial commencement, had a diagnosis of long COVID according to the WHO criteria [39], and reported persistent fatigue, operationalized as the response “most or all the time” to item seven of the Center for Epidemiological Studies Depression Scale (“I felt that everything I did was an effort”) [40]. Candidate participants were excluded if they were intolerant to beetroot juice, reported conditions and/or treatments potentially interfering with trial outcomes (e.g., pregnancy or breastfeeding, diabetes, regular use of steroids or non-steroidal anti-inflammatory drugs, immunosuppressants, nitrate supplementation), or were engaged in other intervention trials for long COVID.

Eligible participants were randomized to receive a daily supplementation of 200 mL beetroot juice (Aureli Mario S.S. Agricola, Ortucchio, Italy; batch #SBRRT140121) [41] or the same volume of placebo for 14 days. The active supplement and the placebo were indistinguishable in appearance and taste. Outcome assessors were blinded to group assignment. Details on the composition of beetroot juice are reported in Supplementary Table S1.

### 4.2. Medical History, Anthropometry, and Functional Measures

Information on comorbid conditions, drug treatments, and time elapsed from COVID-19 diagnosis was collected by self-report and a review of medical charts whenever available. Body height and weight were measured with a standard stadiometer and an analogic medical scale, respectively. The body mass index (BMI, kg/m^2^) was calculated as the ratio between body weight and the square of height.

Physical performance was evaluated by the 6MWT and isometric handgrip strength at baseline and after 14 days of intervention. Both tests were administered by trained personnel according to standardized protocols [42,43]. Briefly, for the 6MWT, participants were asked to walk up and down a 20 m hallway for six min to cover as much distance as possible at their preferred walking speed. The total distance walked (m) was recorded and included in the analysis. Isometric handgrip strength (kg) was measured on the dominant side using a handheld hydraulic dynamometer (North Coast Medical, Inc., Morgan Hill, CA, USA). The test was conducted with participants seated on a chair with the shoulder in a neutral position, the elbow near the trunk and flexed at 90°, and the wrist in a neutral position (thumbs up). The contralateral arm was kept relaxed under the thigh. Participants were instructed to squeeze the handle as strong as they could. A familiarization trial was allowed before the actual testing. 

The FMD test was employed to measure endothelial function [44] using an iU22 2D ultrasound system (Philips Electronics, Amsterdam, The Netherlands) according to standard protocols [44,45,46]. Briefly, the diameter of the brachial artery was measured at baseline and after the abrupt release of a blood pressure cuff that halted the circulation of the forearm by applying a pressure of 250 mmHg for 5 min. FMD was quantified as the percentage increase in arterial diameter following cuff release relative to the baseline diameter.

### 4.3. Blood Collection and Processing

Blood samples were collected at baseline and at 14 days after overnight fasting using standard collection tubes (BD Vacutainer^®^; Becton, Dickinson and Co., Franklin Lakes, NJ, USA). For serum separation, samples were left at room temperature for 30 min and were then centrifuged at 1000× *g* for 10 min at 4 °C. Serum aliquots were stored at −80 °C until analysis. 

### 4.4. Isolation and Characterization of Small Extracellular Vesicles 

#### 4.4.1. Purification of Extracellular Vesicles 

EVs were purified from the serum via ultracentrifugation [47,48,49]. Briefly, serum was diluted with an equal volume of phosphate buffer 1X (PBS1X) and centrifuged at 2000× *g* for 30 min at 4 °C to pellet cell debris. The supernatant was collected and centrifuged at 12,000× *g* for 45 min at 4 °C to eliminate apoptotic bodies and large vesicles. Afterward, the supernatant was ultracentrifuged at 110,000× *g* for 70 min at 4 °C and then filtered through 0.22 µm filters. The obtained pellet was further ultracentrifuged at 110,000× *g* for 70 min at 4 °C and finally resuspended in PBS1X [47]. 

#### 4.4.2. Western Immunoblot Analysis of Extracellular Vesicles

EVs were lysed in a Laemmli buffer (100 mM Tris–HCl pH 6.8, 4% sodium dodecyl sulfate (SDS), 20% glycerol, and 0.2% bromophenol blue) and quantified using the BCA assay (Thermo Fisher Scientific, Waltham, MA, USA) or by Stain-free gels (Bio-Rad, Hercules, CA, USA). Proteins were separated by 12% SDS–polyacrylamide gel electrophoresis and subsequently electroblotted onto polyvinylidene fluoride Immobilon-P membranes (Millipore, Billerica, MA, USA), as previously described [50,51]. Membranes were incubated overnight with primary antibodies and subsequently with anti-mouse and anti-rabbit peroxidase-conjugated secondary antibodies for 1 h at room temperature. The purity of EVs was ascertained according to the guidelines by the International Society for Extracellular Vesicles (ISEV) [48,49]. The presence of Alix, tumor susceptibility gene 101 (TSG101), and of tetraspanins CD9, CD63, and CD81 (positive controls) as well as the absence of the non-EV component ribosomal protein s6 (RPS6) (negative control) was verified. Images were acquired with the ChemiDoc MP Imaging System and analyzed by Image Lab TM software version 6.0.1 (Bio-Rad Laboratories). Protein expression levels were quantified by densitometry and normalized against total protein loadings.

#### 4.4.3. Analysis of Extracellular Vesicles by Transmission Electron Microscopy Imaging

According to ISEV guidelines [48,49], transmission electron microscopy (TEM) was carried out in randomly chosen purified vesicle suspensions to confirm enrichment in EVs. The TEM imaging analysis of isolated vesicles was performed with a JEOL JEM-1011 transmission electron microscope operating at 100 kV (JEOL, Tokyo, Japan) and equipped with a 7.1-megapixel CCD camera (Orius SC1000, Gatan, Pleasanton, CA, USA). TEM image analysis was performed with Gatan Digital Micrograph™ (DM) software. Samples were prepared according to the protocol proposed by Kreger et al. [52] with modifications. Briefly, 5 µL of a concentrated vesicle suspension was dropped on a Formvar-coated copper grid and then infiltrated with a carboxymethyl dextran solution. The resulting ultrathin polysaccharide layer prevented vesicle collapse on the dried grids. Finally, the grids were stained with UranyLess EM Stain (Electron Microscopy Sciences, Hatfield, PA, USA), following the standard protocol provided by the manufacturer.

#### 4.4.4. Measurement of Circulating Cell-Free Mitochondrial DNA by Droplet Digital Polymerase Chain Reaction

ccf-mtDNA was measured in serum by droplet digital polymerase chain reaction (ddPCR), as described by Podlesniy and Trullas [53]. All reagents, consumables, and instruments were purchased from Bio-Rad Laboratories. Twenty μL reaction mixtures were prepared containing 10 μL of 2× ddPCR Supermix for Probes (No-dUTP), 0.9 µM mtDNA forward primer (mtDNA-85F, 5′-CTCACTCCTTGGCGCCTGCC-3′), 0.9 µM mtDNA reverse primer (mtDNA-85R, 5′-GGCGGTTGAGGCGTCTGGTG-3′), and 0.1 µM hydrolysis probe (FAM-mtDNA-85P, 6-carboxyfluorescein (FAM)-50-CCTCCAAATCACCACAGGACTATTCCTAGCCATGCA-30-Black Hole Quencher-1(BHQ-1)). One μL of serum was used as a template source for the ddPCR reaction. The reaction mixture and the ddPCR Droplet Generation Oil for Probes were placed in a DG8 Cartridge for the Droplet Generator and partitioned into droplets using a QX200 Droplet Generator, according to the manufacturer’s instructions to partition samples into around 20,000 nL-sized droplets. The emulsified sample (∼40 µL) was transferred to a 96-well PCR plate and sealed with Pierceable Foil Heat Seal using a PX1 PCR plate sealer. PCR was performed on a T100 Thermal Cycler using standard cycling conditions as follows: 95 °C for 10 min, 40 cycles of 94 °C for 30 s, followed by 60 °C for 1 min, 98 °C for 10 min, then held at 4 °C. The ramp rate between all steps was 2 °C/s. Following the PCR amplification of target mtDNA, fluorescence was measured on a QX200 Droplet Reader detecting, on average, 10,000–20,000 droplets per sample. All assayed samples yielded more than 10,000 droplets. A detection system for FAM was used to analyze each droplet individually, providing a direct quantification of target DNA based on PCR-positive and PCR-negative droplets count. Analysis was performed with Bio-Rad QX Manager 1.2 Standard Edition. The software establishes a threshold for positive droplets based on droplet clustering across all samples that the user can confirm or modify by comparison with a negative, serum-free control. The primary output of the analysis is the concentration of mtDNA copies in starting template per µL. 

#### 4.4.5. Quantification of Mitochondrial DNA Copy Number in Purified Extracellular Vesicles by Droplet Digital Polymerase Chain Reaction

To accurately quantify DNA within EVs, all DNA at the EV surface must be removed. To this aim, Baseline-ZERO DNase (Epicentre—Illumina, Madison, WN, USA) was used according to the manufacturer’s instructions. The reaction was scaled down to be adapted to purified EVs and ddPCR volumes. Briefly, EVs were resuspended in RNase-free water, 10× Baseline-ZERO DNase Reaction Buffer, and 1 MBU of Baseline-ZERO DNase, and incubated at 37 °C for 15 min. Afterward, Baseline-ZERO DNase was inactivated by adding 10× Baseline-ZERO DNase Stop Solution to the sample followed by incubation at 65 °C for 10 min. Then, 2 µg of purified EVs was used as a template source in ddPCR reactions. The mtDNA copy number was then measured as described in the previous section. 

### 4.5. Measurement of Circulating Inflammatory Markers

A panel of selected inflammatory markers including IL-1β, IL-ra, IL-8, IFN- γ, and TNF-α was measured in duplicate in serum samples as part of a larger panel of mediators of the Bio-Plex Pro Human Cytokine 27-plex Assay kit (#M500KCAF0Y, Bio-Rad) on a Bio-Plex^®^ System with Luminex xMap Technology (Bio-Rad). Data acquisition was performed with the Bio-Plex Manager Software 6.1 (Bio-Rad) using instrument default settings. The optimization of standard curves across all the assayed analytes was carried out to remove outliers. IL-6 was assayed using a commercially available kit on an ELLA^TM^ automated immunoassay system (Bio-Techne, San Jose, CA, USA).

### 4.6. Statistical Analysis

Data are shown as mean ± standard deviation (SD) for continuous variables and as absolute numbers and percentages for categorical variables. Data were tested for normality, and some variables exhibited a non-normal distribution. Comparisons of pre- post-intervention variables in the placebo and beetroot juice groups were performed by dependent *t*-test or Wilcoxon test, according to Gaussian distribution. One-way and two-way ANCOVA followed by a Bonferroni post hoc test were used to examine the effects of interventions on the main outcomes, adjusting the analysis for baseline values, and to compare differences between the intervention groups, respectively. ANCOVA was conducted despite the presence of non-parametric data, as assumptions such as linearity, the homogeneity of regression slopes, and the independence of covariates were carefully evaluated and deemed suitable for the analysis. Delta values were determined by calculating the differences between baseline and post-intervention values. Pearson’s and Spearman’s correlation analysis was performed to determine the association between crude and delta values of physical performance, endothelial function, and biomarkers, adjusting according to group allocation. The level of significance was set at alpha 5% (*p* < 0.05), and all analyses were performed using GraphPad Prism 6.0 (San Diego, CA, USA).

## 5. Conclusions

The administration of beetroot juice for 14 days was associated with a quenching of the inflammatory response in adults with long COVID. A negative correlation between vesicular markers of MQC and the performance on the 6MWT as well as FMD was also identified at 14 days independent of the intervention group. These findings suggest that an amelioration of mitochondrial quality, possibly mediated by mitochondria-derived vesicle recycling, may be among the mechanisms supporting improvements in physical performance and endothelial function during the recovery from long COVID.

## Figures and Tables

**Table 1:** Characteristics of study participants at baseline (*n* = 25).

Characteristic	Placebo (*n* = 10)	Beetroot Juice (*n* = 15)	Total (*n* = 25)
Age, years	40.2 (11.5)	38.3 (7.7)	39.1 (9.3)
Women, *n* (%)	6 (60.0)	8 (53.3)	14 (56.0)
Body mass index, kg/m^2^	25.6 (5.5)	24.6 (3.9)	25.0 (4.5)
Hospitalized during acute COVID-19, *n* (%)	0 (0.0)	2 (13.3)	2 (8.0)
Time from COVID-19 diagnosis, days	116.9 (25.4)	112.7 (32.9)	114.4 (29.6)
Comorbid conditions *	2 (20.0)	3 (20.0)	5 (20.0)
Medications	2 (20.0)	3 (20.0)	5 (20.0)
Handgrip strength, kg	30.8 (16.1)	34.8 (9.5)	33.1 (12.5)
6 min walk test, m	558.8 (75.1)	576.6 (56.5)	570.1 (62.7)
Flow-mediated dilation, %	13.2 (6.0)	11.0 (4.8)	11.9 (5.3)

Data are shown as mean (standard deviation) for continuous variables and number (percent) for categorical variables. * include hypertension and thyroid disorders.

## Data Availability

The data presented in this study are available upon request from the corresponding author due to ethics/privacy restrictions pending approval by the Gemelli against COVID Scientific Committee.

## Supplementary Materials

The following supporting information can be downloaded at https://www.mdpi.com/article/10.3390/ijms26031224/s1.
