# Supplementary material for: Mitochondria-Derived Vesicles and Inflammatory Profiles of Adults with Long COVID Supplemented with Red Beetroot Juice: Secondary Analysis of a Randomized Controlled Trial
Source: Int J Mol Sci. 2025 Jan 30;26(3):1224. doi: 10.3390/ijms26031224 (PMC11818272; doi:10.3390/ijms26031224)
Supplement: Supplementary file 1 [file ijms-26-01224-s001.zip › ijms-3434425-supplementary.pdf]

Table S1. Concentrations of inflammatory markers at baseline and after 14 days of intervention in the placebo (*n* = 10) and red beetroot juice (*n* = 15) groups.

Participant ID	Baseline						Post-intervention (14 days)					
	**IL-1β**	**IL-1ra**	**IL-6**	**IL-8**	**IFN-γ**	**TNF-α**	**IL-1β**	**IL-1ra**	**IL-6**	**IL-8**	**IFN-γ**	**TNF-α**
Placebo2	0.30	350.8	3.31	5.27	2.53	42.8	0.30	351.8	1.76	4.19	1.92	36.0
Placebo6	0.88	493.5	0.34	6.04	3.14	45.9	0.51	492.5	0.65	2.97	2.53	39.9
Placebo9	0.88	425.2	2.47	4.49	2.53	46.1	0.30	350.8	2.14	2.67	1.92	36.5
Placebo16	1.22	493.5	0.66	12.36	3.44	47.3	0.51	267.0	2.21	2.37	1.92	39.4
Placebo18	0.88	557.1	2.86	4.49	3.44	44.7	0.88	493.5	1.41	4.80	3.14	44.4
Placebo20	0.69	350.8	1.16	6.04	2.53	35.7	0.51	425.2	0.79	5.42	1.61	30.9
Placebo22	0.51	425.2	1.43	3.42	2.53	50.6	0.69	493.5	1.49	5.73	3.75	40.5
Placebo24	1.17	433.9	4.31	2.78	3.63	62.9	1.17	372.2	2.76	4.92	3.63	63.3
Placebo25	1.04	431.5	0.78	7.70	2.29	63.3	0.52	384.2	0.68	3.85	1.95	56.3
Placebo31	1.72	430.4	1.84	8.04	4.61	67.4	1.04	383.2	0.79	5.97	1.95	57.6
Beetroot1	0.88	493.5	0.67	21.77	2.53	38.2	0.69	267.0	0.78	2.37	1.61	33.7
Beetroot4	0.69	425.2	0.69	6.35	2.53	41.6	0.51	425.2	0.63	3.58	3.14	40.5
Beetroot7	0.88	493.5	0.69	4.19	2.53	43.9	0.30	350.8	0.71	3.58	2.83	29.5
Beetroot10	1.05	557.1	1.25	9.50	3.44	46.1	0.60	425.2	0.89	1.77	2.53	39.9
Beetroot11	0.88	425.2	2.28	8.55	3.14	47.5	0.30	425.2	0.32	4.20	2.83	41.3
Beetroot13	1.22	425.2	1.15	6.51	3.44	46.1	0.79	350.8	0.73	2.97	2.53	31.4
Beetroot15	0.96	425.2	1.46	5.42	4.66	39.9	0.51	425.2	1.39	3.88	3.44	37.1
Beetroot17	0.88	350.8	1.75	4.34	2.53	41.1	0.69	350.8	1.18	3.58	2.68	34.0
Beetroot19	0.07	350.8	0.78	2.67	2.38	40.2	0.07	350.8	1.43	3.27	2.83	34.3
Beetroot26	0.88	425.2	0.88	5.42	1.92	41.9	0.07	350.8	0.53	4.19	2.83	32.6
Beetroot27	1.04	430.9	1.29	8.04	1.95	64.1	0.92	387.1	1.21	5.09	1.95	66.6
Beetroot28	1.54	423.8	0.68	10.78	3.96	73.0	1.23	388.4	1.43	5.62	1.95	62.5
Beetroot29	0.92	436.3	1.86	2.78	1.25	65.4	0.66	380.0	2.32	3.50	1.95	61.7
Beetroot30	0.92	433.8	1.80	6.31	0.89	67.4	0.59	376.9	0.93	5.27	1.25	58.5
Beetroot32	0.66	431.7	0.65	6.66	1.95	69.4	0.92	378.9	0.69	7.35	1.95	63.3

Values are pg/mL. Abbreviations: IFN-γ, interferon gamma; IL, interleukin; IL-1ra, interleukin 1 receptor antagonist; TNF-α, tumor necrosis factor.

Table S2. Concentrations of metabolites contained in the red beetroot juice supplement used in the study.

Class	Metabolites	Concentration (mg/100mL)
Amino acids	Alanine	90.70 ± 7.00
	Asparagine	28.97 ± 2.25
	Gamma-aminobutyric acid	11.95 ± 0.88
	Glutamate	155.90 ± 6.80
	Glutamine	80.20 ± 8.40
	Isoleucine	15.9 ± 1.20
	Leucine	7.81 ± 0.59
	Phenylalanine	0.57 ± 0.05
	Threonine	10.78 ± 0.86
	Tryptophan	2.65 ± 0.19
	Tyrosine	6.09 ± 0.47
	Valine	15.40 ± 1.20
Organic acids	Betalamic acid	0.76 ± 0.07
	Citrate	169 ± 14
	Formate	1.42 ± 0.09
	Fumarate	0.69 ± 0.05
	Malate	84.56 ± 7.25
	4-hydroxybenzoate	0.39 ± 0.03
	4-hydroxycumarate	1.05 ± 0.05
Carbohydrates	Arabinose	9.56 ± 0.78
	Fructose	161 ± 11
	Glucose	393 ± 31
	Sucrose	5610 ± 454
Miscellaneous	Betaine	245 ± 20
	Betanin	12.55 ± 1.12
	Choline	1.91 ± 0.18
	Dopamine	7.26 ± 0.65
	Myoinositol	147 ± 12
	Trigonelline	1.13 ± 0.11

Values are mean ± standard deviation. Metabolites were measured by nuclear magnetic resonance spectroscopy.
